# Adverse Events of Pirfenidone for the Treatment of Pulmonary Fibrosis: A Meta-Analysis of Randomized Controlled Trials

**DOI:** 10.1371/journal.pone.0047024

**Published:** 2012-10-09

**Authors:** Chunguo Jiang, Hui Huang, Jia Liu, Yanxun Wang, Zhiwei Lu, Zuojun Xu

**Affiliations:** 1 Department of Respiratory Medicine, Peking Union Medical College Hospital, Chinese Academy of Medical Sciences & Peking Union Medical College, Beijing, China; 2 Department of Respiratory Medicine, Yijishan Hospital of Wannan Medical College, Wuhu, China; Tehran University of Medical Sciences, Islamic Republic of Iran

## Abstract

**Background:**

Pirfenidone (PFD) is a novel antifibrotic agent approved for patients with pulmonary fibrosis. However, there are concerns regarding toxicity of the drug. In this meta-analysis, we analyzed the adverse events (AEs) of PFD for the treatment of pulmonary fibrosis.

**Methods:**

We performed a systematic search of PubMed, Embase, ClinicalTrials.gov, and Cochrane Central Register of Controlled Trials for trials published between January 1999 and October 2011. Data extracted from literature were analyzed with Review manager 5.0.24.

**Results:**

The results of six randomized controlled trials (1073 participants) revealed that the number of individuals who discontinued PFD therapy was significantly higher than patients receiving placebo. The PFD group had a significantly higher rate of gastrointestinal (nausea, dyspepsia, diarrhea, and anorexia), neurological (dizziness and fatigue), and dermatological (photosensitivity and rash) AEs compared to the placebo group.

**Conclusions:**

PFD used for the treatment of pulmonary fibrosis is not so safe or well-tolerated. Notably, gastrointestinal, neurological and dermatological adverse effects were more common in patients receiving PFD therapy, and therefore appropriate precaution is needed.

## Introduction

Idiopathic pulmonary fibrosis (IPF) is a chronic fibrosing interstitial lung disease of unknown etiology that is clinically characterized by progressive worsening of dyspnea and lung function, and the 3- and 5-year mortality rates at approximately 50% and 80%, respectively, in the absence of lung transplantation [1], [2]. To date, the results of pharmacologic trials for treating IPF have been disappointing. Interferon gamma [3], bosentan [4], etanercept [5], and sildenafil [6] have all failed to demonstrate benefits in placebo-controlled trials. High-dose N-acetylcysteine showed a modest but significant effect on the preservation of forced vital capacity (FVC) in patients receiving a combination therapy with prednisone and azathioprine compared to patients randomized to prednisone and azathioprine only [7]. These results underscore the need for the development of novel therapies that are efficacious for treating IPF.

Pirfenidone (PFD) has been proposed as an intriguing antifibrotic agent for use in this difficult-to-treat population, and has received approval in Japan, India, and Europe. PFD was found to attenuate bleomycin-induced lung fibrosis in hamsters [8], [9], and was able to scavenge reactive oxygen species (ROS) *in vitro* [10]. The initial human data supporting a role for PFD in the treatment of IPF was first published by Raghu *et al*. in 1999 [11]. Pooled data from later studies supported a treatment effect on FVC, progression-free survival, and Six-Minute Walk Distance (6MWD) [12]–[17]. However, some problems have been associated with PFD therapy, and a number of patients discontinue the drug because of adverse events (AEs), such as nausea and photosensitivity. Therefore, the safety of PFD in this patient population is still uncertain. In this study we conducted a meta-analysis of available published literature to assess adverse reactions and tolerability of PFD versus placebo for the treatment of pulmonary fibrosis in clinical use.

**Figure 1:**
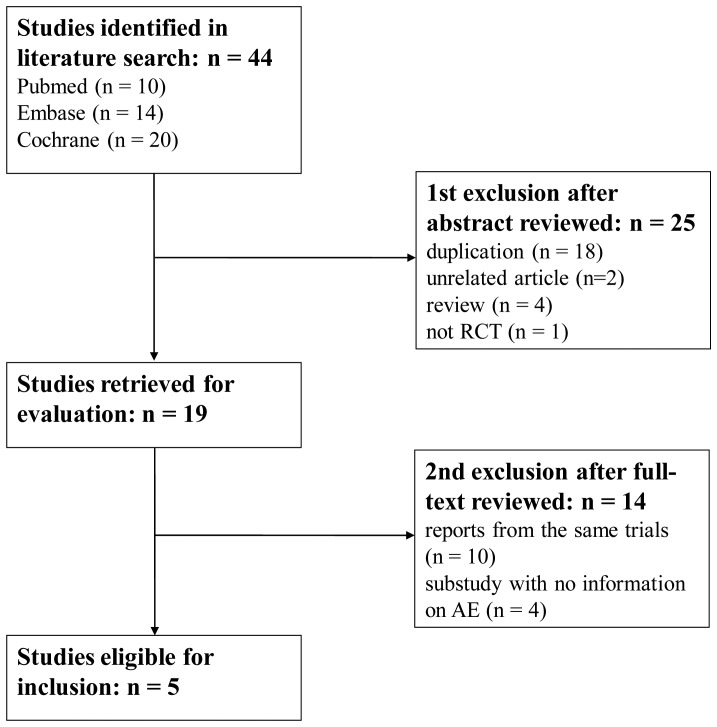
Flow diagram of assessment of studies identified in the meta-analysis. AE = Adverse event; RCT = Randomized controlled trial.

## Materials and Methods

### Data Collection

The aim of this meta-analysis was to include all publicly available data on the treatment of pulmonary fibrosis with PFD from randomized controlled trials (RCTs). Two authors performed systematic searches of the medical literature to identify articles from PubMed, Embase, ClinicalTrials.gov, and Cochrane Central Register of Controlled Trials that had been published between January 1999 and October 2011 according to a standardized protocol. These studies were combined using the set operator and with papers that included a list of relevant keywords for disease (pulmonary fibrosis or lung fibrosis) and treatment (pirfenidone). The searches were not restricted to English language literature. In addition, we searched reference lists and conference abstracts by hand, and checked relevant reviews and book chapters. Efforts also were made to contact the investigators for finding additional unpublished studies.

**Figure 2:**
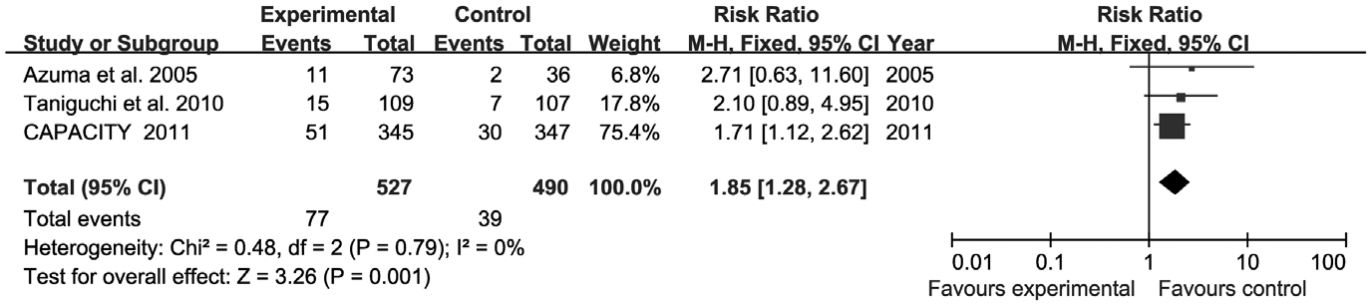
Forest plot of trials of PFD versus placebo examining the effect on relative risk of withdrawal of therapy due to AEs. AE = Adverse event; df = degrees of freedom; M-H = Mantel-Haenszel.

**Figure 3:**
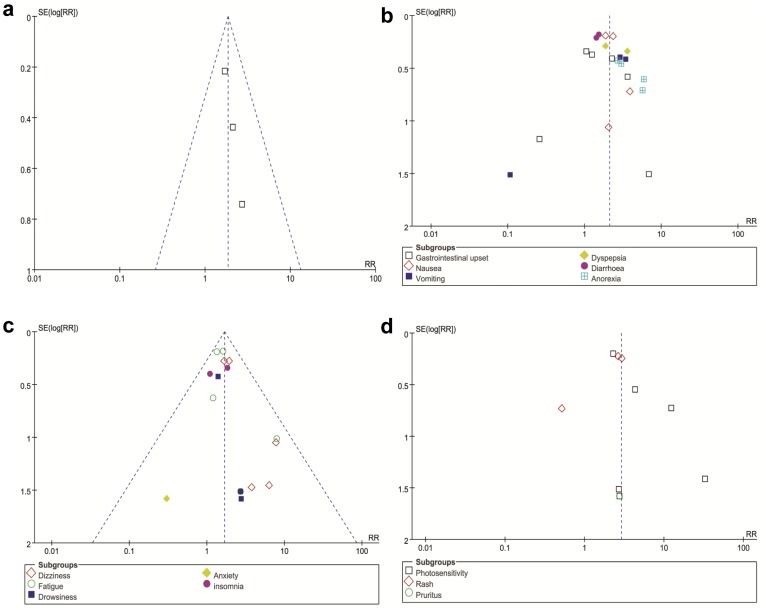
Funnel plot to assess for evidence of publication bias. a. Funnel plot for the studies on withdrawal of therapy due to AEs; b. Funnel plot for the studies on gastrointestinal AEs; c. Funnel plot for the studies on neurological AEs; d. Funnel plot for the studies on dermatological AEs. See Figure 2 legend for expansion of abbreviations.

### Study Selection

Paired reviewers (CJ and HH) independently evaluated references for eligibility using a two-stage procedure. In the first stage, all identified abstracts were evaluated for appropriateness to the study question. All potentially relevant studies were retrieved and selected for full-text review to determine whether or not they met all eligibility criteria in the second stage. Articles that were selected by either reviewer were assessed, and the inclusion and exclusion criteria were evaluated by both reviewers in the second stage. Any disagreements were resolved by discussion.

**Figure 4:**
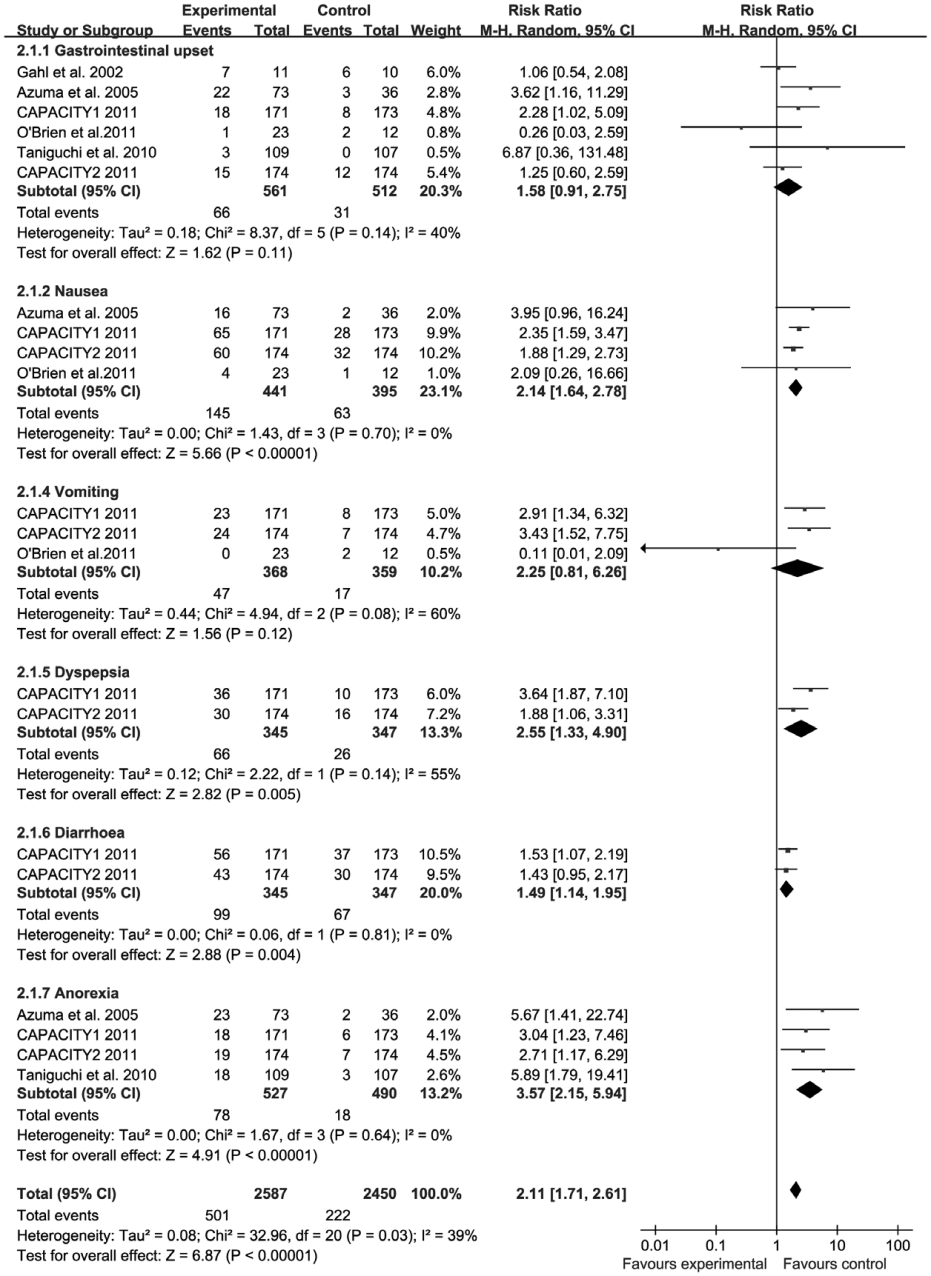
Forest plot of trials of PFD versus placebo examining the effect on relative risk of gastrointestinal AEs. See Figure 2 legend for expansion of abbreviations.

#### Eligibility criteria

(1) Type of participants: adults with pulmonary fibrosis, include idiopathic pulmonary fibrosis, Hermansky-Pudlak syndrome, pulmonary fibrosis associated with connective tissue diseases, and pulmonary fibrosis caused by drug, radiation or other disease. Studies involving patients who received PFD for other diseases were excluded. (2) PFD dose: the dose of PFD had to be ≥1800 mg per day. (3) Outcome measures were adverse events. After extraction, three categories (gastrointestinal, neurological, dermatological adverse events) were of the most interest to us. (4) Type of publication: only full papers on original patient data reporting AE of PFD treatment were considered for further appraisal. (5) Type of study: studies had to be RCTs comparing PFD with placebo or with other anti-fibrosis drugs.

**Figure 5:**
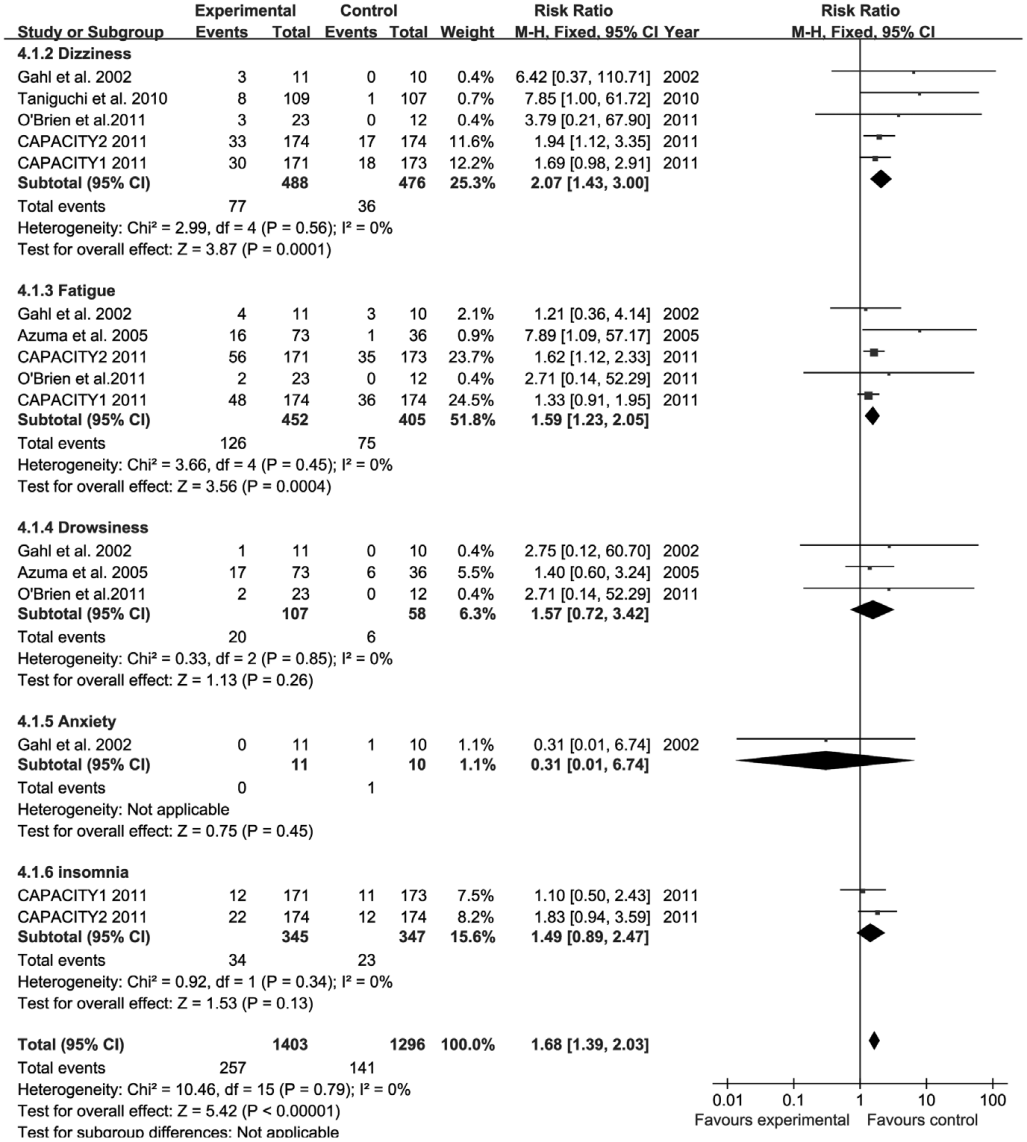
Forest plot of trials of PFD versus placebo examining the effect on relative risk of neurological AEs. See Figure 2 legend for expansion of abbreviations.

### Data Extraction and Quality Assessment

Data concerning the type and number of AEs were extracted onto specially developed forms by two reviewers, and then the verified data were entered onto a Microsoft Excel spreadsheet (XP professional edition; Microsoft Corp, Redmond, WA, USA). Trial characteristics, including the setting (area of origin, disease, and phase), proportion of male patients, mean age of included patients, and dosage schedule of PFD therapy were recorded to allow exploration of potential reasons for any heterogeneity detected between trial results. No attempt was made to include unpublished data. We assessed methodological quality using the Jadad score [18]. We graded each parameter of trial quality as full mark (5), high mark (≥3), or low mark (≤2).

**Figure 6:**
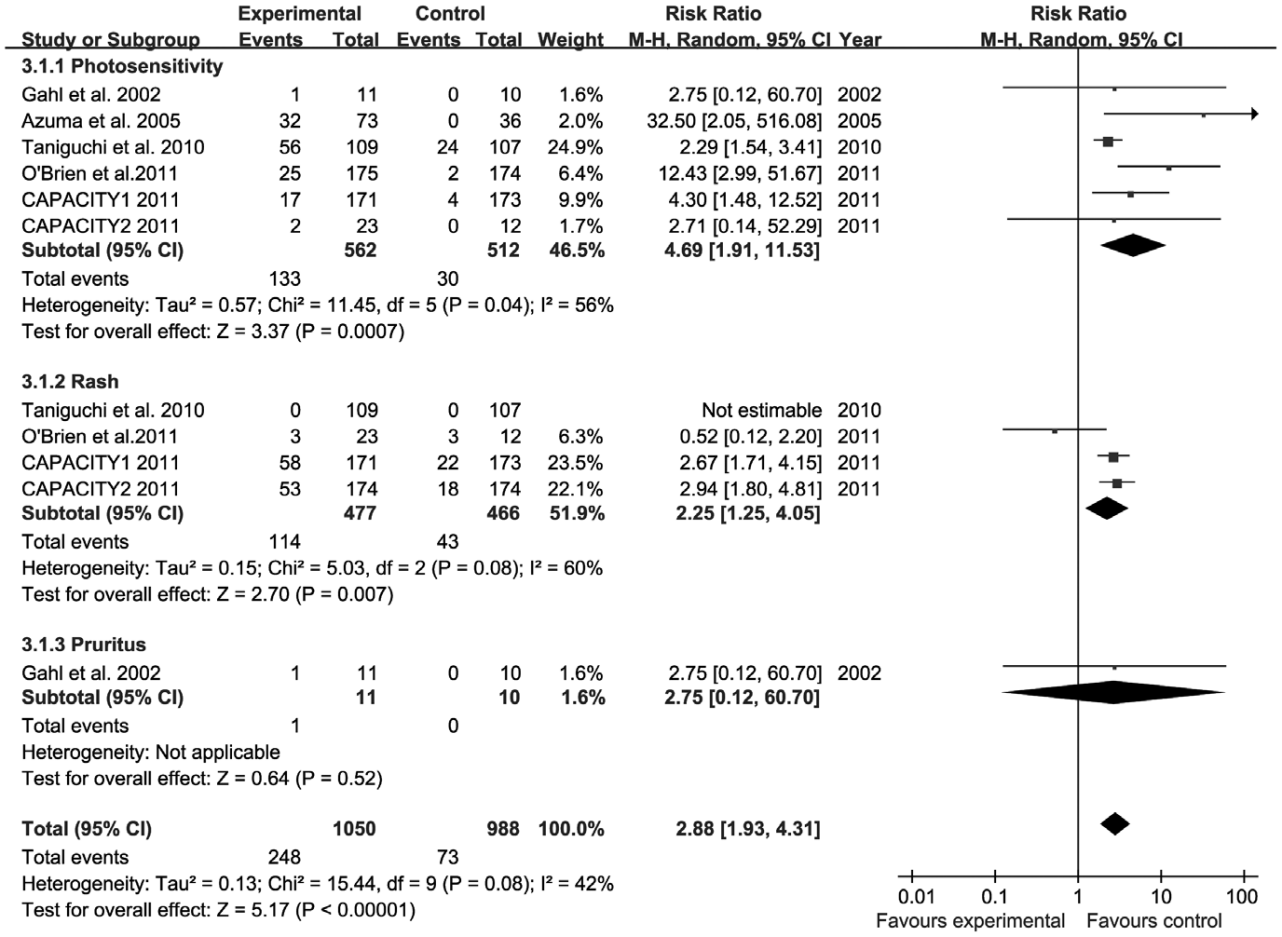
Forest plot of trials of PFD versus placebo examining the effect on relative risk of dermatological AEs. See Figure 2 legend for expansion of abbreviations.

### Data Synthesis and Analysis

We analyzed the forest plots using the Review Manager 5.0.24 statistical software (Cochrane Collaboration, Oxford, UK). As dichotomous outcomes, the impact of PFD therapy on the incidence of total and specific individual adverse effects versus the comparison regimen was expressed as a combined relative risk (RR) with a 95% confidence interval (CI). The fixed-effect model weighted by the Mantel-Haenszel method was used, and the random effect model was used in the case of significant heterogeneity (*P* value of χ^2^ test <0.05 and *I^2^*>50%). A funnel plot test was used to assess for evidence of publication bias. A *P*<0.05 was considered statistically significant.

## Results

### Study Identification

The process of identifying eligible RCTs is summarized in Figure 1. Using the search strategy, 44 studies were retrieved. After title and abstract evaluation, 19 studies were used for further assessment. After a full-text review, six trials (five articles) were included in the meta-analysis and AE data were extracted from these studies.

Detailed characteristics of the included RCTs are provided in Table S1. The six RCTs [12]–[16] enrolled a total of 1073 participants (561 in the PFD group and 512 in the placebo group) published between 2002 and 2011. Two of the trials were conducted in America [12], [15], two in Japan [13], [14], and two were multi-national studies [16]. In addition, two studies assessed treatment of Hermansky-Pudlak syndrome (HPS) and the remaining trials assessed treatment of IPF. The total daily dose of PFD ranged from 1800 mg to 2403 mg, and all comparison regimens were placebo. The number of participants in each RCT ranged from 21 to 348 individuals. The proportion of male patients varied between 40 and 94%, and the mean age of individuals ranged from 34.0 to 67.0 y.

The Jadad score of all studies surpassed four points, where four were graded as full mark and two were graded as high mark (Table S1). All studies were double-blind, randomized, and placebo controlled, and all included a description of withdrawals and drop outs [12]–[16]. Four of the studies reported the method used to generate the randomization schedule [13], [14], [16]. One of the studies recorded AEs using a questionnaire [12], and another used diary cards [15]. The remainder of the trials did not state how they collected AE data [13], [14], [16].

### Withdrawal of Therapy Due to AEs from PFD Versus Placebo

Four trials reported the overall rates of discontinuation from therapy due to AEs from PFD versus placebo [13], [14], [16]. Withdrawal of therapy due to AEs was significantly more common in the PFD group than the placebo group for patients with pulmonary fibrosis (RR = 1.85, 95% CI: 1.28–2.67, *P* = 0.001). The heterogeneity test was not substantial, as assessed by the *I^2^* statistics (Q (d.f. = 2) = 0.48, *P* = 0.79, *I^2^* = 0%) (Figure 2). Publication bias was evaluated using the funnel plot (Figure 3a). There was no evidence to suggest publication bias according to the relative symmetry in the funnel plot of studies on withdrawal of therapy due to AEs.

### Number of Gastrointestinal AEs from PFD Versus Placebo

All trials reported the number of individuals experiencing gastrointestinal AEs from PFD versus placebo [12]–[16]. The combined results of the six trials revealed that the PFD group had a significantly higher rate of gastrointestinal AEs compared to the placebo group (RR = 2.11, 95% CI: 1.71–2.61, *P*<0.001). The heterogeneity test was substantial, as assessed by the *I^2^* statistics (Q (d.f. = 20) = 32.96, *P* = 0.03, *I^2^* = 39%) (Figure 4). A sub-group analysis of gastrointestinal AEs, including gastrointestinal upset, nausea, vomiting, dyspepsia, diarrhea, and anorexia is described. Nausea (RR = 2.14, 95% CI: 1.64–2.78, *P*<0.001), dyspepsia (RR = 2.55, 95% CI: 1.33–4.90, *P* = 0.005), diarrhea (RR = 1.49, 95% CI: 1.14–1.95, *P* = 0.004), and anorexia (RR = 3.57, 95% CI: 2.15–5.94, *P*<0.001) all occurred significantly more frequently in patients treated with PFD compared to patients receiving placebo. However, PFD was not associated with a more significant increase in gastrointestinal upset (RR = 1.58, 95% CI: 0.91–2.75, *P* = 0.11) and vomiting (RR = 2.25, 95% CI: 0.81–6.26, *P* = 0.12). Publication bias was not evident, as estimated by funnel plot for the studies on gastrointestinal AEs (Figure 3b).

### Number of Neurological AEs from PFD Versus Placebo

All trials reported the number of individuals experiencing neurological AEs from PFD versus placebo [12]–[16]. The combined results of the six trials revealed that the PFD group had a significantly higher rate of neurological AEs compared to the placebo group (RR = 1.68, 95% CI: 1.39–2.03, *P*<0.001). The heterogeneity test was not substantial, as assessed by the *I^2^* statistics (Q (d.f. = 15) = 10.46, *P* = 0.79, *I^2^* = 0%) (Figure 5). A sub-group analysis of neurological AEs, including dizziness, fatigue, drowsiness, insomnia, and anxiety is described. Dizziness (RR = 2.07, 95% CI: 1.43–3.00, *P*<0.001) and fatigue (RR = 1.59, 95% CI: 1.23–2.05, *P*<0.001) occurred significantly more frequently in patients treated with PFD compared to patients receiving placebo. However, PFD was not associated with a more significant increase in drowsiness (RR = 1.57, 95% CI: 0.72–3.42, *P* = 0.26) and insomnia (RR = 1.49, 95% CI: 0.89–2.47, *P* = 0.13). Only one trial reported anxiety, and the data were not sufficient to pool [12]. Funnel plot for the studies on neurological AEs was relatively symmetrical (Figure 3c), and publication bias was not evident.

### Number of Dermatological AEs from PFD Versus Placebo

All trials reported the number of individuals experiencing dermatological AEs from PFD versus placebo [12]–[16]. The combined results of the six trials revealed that the PFD group had a significantly higher rate of dermatological AEs compared to the placebo group (RR = 2.88, 95% CI: 1.93–4.31, *P*<0.001). Heterogeneity test was not substantial, as assessed by the *I^2^* statistics (Q (d.f. = 9) = 15.44, *P* = 0.08, *I^2^* = 42%) (Figure 6). A sub-group analysis of dermatological AES, including photosensitivity, rash, and pruritus is described. Photosensitivity (RR = 4.69, 95% CI: 1.91–11.53, *P*<0.001) and rash (RR = 2.25, 95% CI: 1.25–4.05, *P* = 0.007) occurred significantly more frequently in patients treated with PFD compared patients receiving placebo. Only one trial reported pruritus, and therefore these data were not sufficient to pool [12]. There was no evidence to suggest publication bias, as estimated by funnel plot for the studies on dermatological AEs (Figure 3d).

## Discussion

To the best of our knowledge, this is the first meta-analysis to examine the safety profile of PFD for treating pulmonary fibrosis. The findings of this study are timely and of critical importance, since there have been previous concerns surrounding the issue of potential AEs from PFD use, which has led to withdrawal of the drug. In this meta-analysis, the number of individuals who discontinued PFD therapy was significantly higher than patients receiving placebo. In addition, the combined results of the six trials revealed that the PFD group had a significantly higher rate of gastrointestinal (nausea, dyspepsia, diarrhea, and anorexia), neurological (dizziness and fatigue), and dermatological (photosensitivity and rash) AEs compared to the placebo group.

Withdrawal of therapy due to AEs was significantly more common in the PFD group, although all PFD-related AEs resolved after discontinuation of the drug. Based on the available evidence, PFD appears to be not so safe or well-tolerated in patients with IPF. To examine the long-term safety of PFD in patients with IPF, an open-label extension study of the CAPACITY trials, called the RECAP study, is currently ongoing. Interim data demonstrated that PFD has a favorable long-term safety profile and generally well-tolerated in patients with IPF [19]; however, because these results have not been published, the full data are not available for review at this time.

The most frequently reported adverse effects from PFD treatment were gastrointestinal, neurological, and dermatological effects. The application of PFD therapy seemed to be more common with these adverse effects. PFD-related side effects, and particularly gastrointestinal events, appear to be more frequent at higher doses and can be improved by dose reductions and by administering PFD with food [20], [21]. In addition, photosensitivity reactions can be minimized by using protective sun creams and avoiding exposure to direct sunlight.

There are several limitations for this study. First, although it has provided data from more than 1000 patients, the number of incorporated studies was still small, and therefore these results may be affected by publication bias. Second, heterogeneities between studies can confuse meta-analysis outcomes and may come from the different basic values and drug doses. Third, there were other frequently reported adverse effects, such as appetite loss [22] and liver enzyme elevation, which were not described and analyzed in detail in this article. Therefore, additional RCTs would be helpful by providing more convincible data to investigate the accuracy of this conclusion.

In conclusion, this meta-analysis shows that PFD used for the treatment of IPF is not so safe or well-tolerated. Notably, gastrointestinal, neurological, and dermatological adverse effects were significantly more common in patients receiving PFD therapies compared to placebo and therefore appropriate precautions are needed. However, if PFD gains additional approval as a therapeutic agent for treating IPF in other countries, then the regulatory authorities will most likely mandate close post-marketing surveillance of AEs. This will be important if less common side effects of PFD are to be recognized. Given the unmet medical need and efficacy results, PFD has a clear role in the treatment of IPF.

## Supporting Information

Table S1 Characteristics of included randomized controlled trials.
(DOC)
