# Supplementary material for: Adverse Events of Pirfenidone for the Treatment of Pulmonary Fibrosis: A Meta-Analysis of Randomized Controlled Trials
Source: PLoS One. 2012 Oct 9;7(10):e47024. doi: 10.1371/journal.pone.0047024 (PMC3467250; doi:10.1371/journal.pone.0047024)
Supplement: Table S1 — Characteristics of included randomized controlled trials. (DOC) [file pone.0047024.s001.doc]

**Table S1.** Characteristics of included randomized controlled trials.

| Study | Location | Disease | Phase | Drug (mg/d) | N | Male | Age (y) | JC |
| --- | --- | --- | --- | --- | --- | --- | --- | --- |
| Gahl et al.  2002 | America | HPS | Ⅱ | 2400  placebo | 11  10 | 5  4 | 41.5 ± 12.1  34.0 ± 9.2 | 4 |
| Azuma et al.  2005 | Japan | IPF | Ⅱ | 1800  placebo | 73  36 | 62  33 | 64.0 ± 7.1  64.3 ± 7.6 | 5 |
| Taniguchi et al.  2010 | Japan | IPF | Ⅲ | 1800  placebo | 109  107 | 85  81 | 65.4 ± 6.2  64.7 ± 7.3 | 5 |
| O'Brien et al.  2011 | America | HPS | Ⅱ | 2403  placebo | 23  12 | 8  6 | 39.2 ± 10.8  43.4 ± 7.7 | 4 |
| CAPACITY1  2011 | Multi-national | IPF | Ⅲ | 2403  placebo | 171  173 | 123  124 | 66.8 ± 7.9  67.0 ± 7.8 | 5 |
| CAPACITY2  2011 | Multi-national | IPF | Ⅲ | 2403  placebo | 174  174 | 118  128 | 65.7 ± 8.2  65.7 ± 8.2 | 5 |

Data are mean ± standard error of the mean (SEM).

N: Number of pairwise comparisons

JC: Jaded score
